# Epidemiological analysis of the Eyam plague outbreak of 1665–1666

**DOI:** 10.1098/rspb.2016.0618

**Published:** 2016-05-11

**Authors:** Lilith K. Whittles, Xavier Didelot

**Affiliations:** Department of Infectious Disease Epidemiology, Imperial College London, London, UK

**Keywords:** plague, interhuman transmission, rodent reservoir, Bayesian analysis, Monte Carlo Markov chain, two-level mixing model

## Abstract

Plague, caused by the bacterium *Yersinia pestis*, is one of the deadliest infectious diseases in human history, and still causes worrying outbreaks in Africa and South America. Despite the historical and current importance of plague, several questions remain unanswered concerning its transmission routes and infection risk factors. The plague outbreak that started in September 1665 in the Derbyshire village of Eyam claimed 257 lives over 14 months, wiping out entire families. Since previous attempts at modelling the Eyam plague, new data have been unearthed from parish records revealing a much more complete record of the disease. Using a stochastic compartmental model and Bayesian analytical methods, we found that both rodent-to-human and human-to-human transmission played an important role in spreading the infection, and that they accounted, respectively, for a quarter and three-quarters of all infections, with a statistically significant seasonality effect. We also found that the force of infection was stronger for infectious individuals living in the same household compared with the rest of the village. Poverty significantly increased the risk of disease, whereas adulthood decreased the risk. These results on the Eyam outbreak contribute to the current debate on the relative importance of plague transmission routes.

## 1. Introduction

Plague, caused by the bacterium *Yersinia pestis*, has been one of the most deadly infectious diseases throughout human existence. Historically, the term has been used to refer to many human calamities, and the bacterium has been implicated in three worldwide pandemics [1,2]. The Justinian Plague of 541–767 is thought to have killed 40–50% of the population and contributed to the decline and fall of the Roman Empire [3,4]. In the fourteenth century, the Black Death ravaged Europe, reportedly killing 25 million people [5]. The third pandemic started in the mid-nineteenth century and lasted a century, focusing mostly on China and India, but spreading also to other continents [1,2]. The once debated question of the causative agent of the Black Death has been confirmed beyond doubt by the identification of *Y. pestis* DNA from victim remains [6–8], and likewise for the Justinian Plague [9,10].

Despite the commonly held view of plague as a historical disease, the bacterium is still present in wild animal reservoirs around the world, and human cases are frequently reported in African and South American countries [11–13]. *Yersinia pestis* is considered to be a potential bioterrorism agent [14,15], and indeed the first recorded use of a biological weapon was during the siege of Caffa in 1346 when the Mongol army catapulted plague-infected corpses over the Crimean city's walls [16]. Public health concern is further increased by sporadic reports of antimicrobial resistance in *Y. pestis* [17,18].

Plague is a zoonosis, primarily found in rodents, although most mammals can be infected [19]. Transmission of *Y. pestis* to humans can occur through contact with infected animals and their parasites. The oriental rat flea, *Xenopsylla cheopis*, is known to be a very effective vector of plague: upon infection its digestive system becomes ‘blocked’, causing vomiting of bacteria into subsequent biting targets [2]. For this reason, rodent-to-human transmission has long been considered the main route of infection. However, human-to-human transmission may be more important than previously thought, via ectoparasites such as lice [20] and the human flea *Pulex irritans* [21]. *Yersinia pestis* was recently found in human fleas collected from plague-affected villages of Tanzania and Madagascar [22,23]. Human fleas do not become blocked in the way rat fleas do, but unblocked fleas are also able to transmit the infection [24]. Interhuman transmission of plague can also occur directly via aerosols following the development of pneumonia [25]. Pneumonic plague is known to progress more quickly and is more frequently fatal, but transmission via this route is thought to be incompatible with historical accounts of the plague [26,27].

The plague outbreak that lasted from September 1665 until October 1666 in the Derbyshire village of Eyam is infamous, not only for its high death toll, but also due to the heroism of the villagers who endured a quarantine and successfully prevented the spread of the disease to neighbouring parishes [28–31]. Historically, the introduction of the *cordon sanitaire* has been considered remarkable, foremost as an act of altruism by the villagers under the direction of the rector William Mompesson and previous incumbent Thomas Stanley, and further because similar contemporary public health measures were unpopular and often disobeyed [32]. The narrative of human tragedy that attaches itself to Eyam is heightened by the suggestion that the quarantine itself prolonged the epidemic and exacerbated the human death toll [30,32]. Furthermore, revisionist historians have questioned whether the quarantine was truly a self-imposed sacrifice, suggesting that the Earl of Devonshire's agreement to furnish provisions may have been tied to the closure of the village [33].

The Eyam outbreak has accordingly been an often-mentioned epidemiological case study, and the village itself a popular tourist attraction. However, there have only been a few attempts to model the epidemiology of the Eyam plague [34–36]. These previous studies have relied on local historian William Wood's account of the village's demography, who, writing about a century after the event, placed the population at around 350 people and the mortality rate at close to 75% [28]. This figure has been disputed based on parish records and hearth tax returns, suggesting a parish population between 850 and 1000 people [37]. This higher estimate would be compatible with an estimate of 750 adults in 1676 [38] and the lack of long-term demographic effect on the Eyam population [39]. A full transcript of the Eyam parish register between 1630 and 1700 [40] combined with the 1664 Eyam hearth tax return has revealed the history of the survivors, leading to a conservative estimate of about 700 people for the population of Eyam at the start of the outbreak [31].

The first proposed model of the Eyam plague [34,35] is a typical example of the susceptible–infectious–removed (SIR) compartmental model and is often mentioned as a case study [41,42]. Infection was assumed to be transmitted directly from human to human and to last exactly 11 days before death. Only the second half of the outbreak was studied, because the SIR model could not explain the first phase. The use of a model that ignores the zoonotic nature of the disease altogether has been noted [27,43], and other acknowledged shortfalls of the model include the exclusion of a latency period between catching plague and becoming infectious and the assumption of perfect mixing between villagers. By contrast, a complex compartmental model with 38 set parameters has more recently been proposed [36], which considered human, rat and flea population dynamics, but assumed perfect mixing of a population with an underestimated size of 350 people [28] and no latent period of infection.

The lack of reliable data on parasites and rodent population dynamics in seventeenth-century England lead us to adopt a more parameter-efficient model. We propose a stochastic compartmental model, considering both rodent-to-human and human-to-human transmission of plague, that incorporates a latent period of infection and allows for an increased rate of human-to-human transmission among members of the same household. An epidemiological Bayesian approach [44] is taken, and the lack of data on infection times and when plague victims became infectious is approached using data augmentation techniques [45]. This allows estimation of the parameters of our model, the relative importance of transmission routes, the role of the household structure and the risk factors of infection. The combination of more detailed data with a novel model, enables us to shed new light on the transmission of the Eyam plague outbreak of 1665–1666, which feeds directly into the debate surrounding the epidemiology of historical plague.

## 2. Results

### (a) Data collection and summary

The Reverend William Mompesson, who was the rector of Eyam at the time of the plague, recorded in the Eyam parish register the names of all victims of the plague and their dates of burial from the first case on 6 September 1665 to the last on 1 November 1666. Although the initial population of Eyam was originally thought to be around only 350 [28], it has since been suspected that the total may in fact be significantly higher [37]. The publication of a meticulously curated version of the Eyam parish register between 1630 and 1700 has confirmed that the initial population was around 700 people [31,39,40]. The register records the gender, date of baptism and date of burial. The register further provides an indicator of whether each death was from plague or other causes, as marked by a later rector, Joseph Hunt, who copied the entire text in the latter half of the seventeenth century.

The hearth tax record for Eyam in 1664 includes details of both taxed and untaxed households, and this was combined with the Eyam parish register to reconstruct the household structure for all persons living in the parish during the time of the plague [31]. The Eyam Museum provides this collated data, as well as additional information such as the approximate year of birth where the exact date of baptism is unknown, and the year of last mention in records (for example, as the beneficiary of a will, a marriage certificate or the birth of a child) where the exact date of death is unknown [46]. All of these data were checked for consistency with the parish register [40] and digitalized to produce the dataset analysed here, which is contained in electronic supplementary material, table S1.

### (b) Exploratory data analysis

Of the 700 people reasonably assumed to have been living in Eyam at the outbreak of plague, 11 are recorded as having died due to causes other than plague during the epidemic, and are as such excluded from the analysis. Infants born during the plague are also excluded from the analysis. This leaves a total population of *N* = 689 people at risk, divided between *M* = 210 households. Out of this total, 257 people died of plague (37%) and 432 (63%) survived it. It is assumed that death from plague occurred on the day prior to recorded burial.

The data were summarized, and Fisher's exact tests were performed to ascertain whether gender, wealth and prior infection in the same household were significant factors in describing the epidemic (table 1). This exploratory analysis showed that household structure and the relative wealth of households were probably important determinants of the epidemic; however, gender was not found to be a significant factor, in agreement with past studies of Eyam [32,37,39].

**Table 1.** Exploratory analysis of the Eyam data using Fisher's exact tests.

quality of interest	factor level	plague victims	survivors	total	*p*-value	significance
gender	male female unknown	133 122 2	211 221 —	344 343 2	0.1308	n.s.
hearth tax	taxed untaxed	52 205	149 283	201 488	<0.0001	extremely
age	under 18 over 18 unknown	116 126 15	160 258 14	276 384 29	0.0136	weakly
prior infection in household	true false	154 103	102 330	256 433	<0.0001	extremely

**Figure 1.**
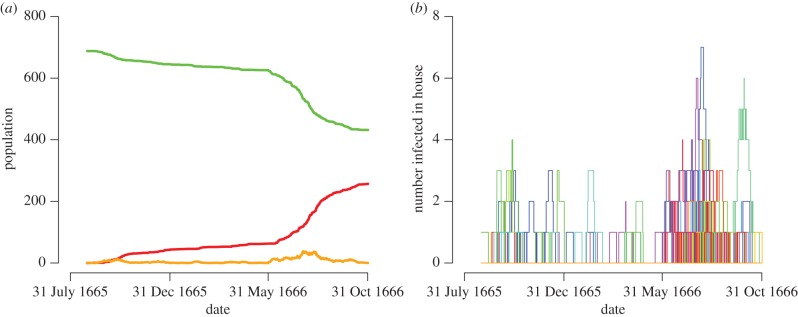
Eyam epidemic plot assuming an 11-day infection period. (*a*) Green line shows susceptible population; orange line shows infected population and red line shows number of deceased. (*b*) Each coloured line represents the number of infected people in a household. (Online version in colour.)

The progression of the epidemic was plotted over time by inferring the number of susceptible and infected villagers using the naive assumption of a fixed 11-day infection period before death, as employed in previous modelling studies [34] (figure 1). Considering the inferred number of infected members at any time-point in each household, the household structure of infection suggested in the exploratory data analysis is evident (figure 1). The epidemic can be described as being made of three periods: the initial peak in October 1665, followed by a period of relative abatement over the winter, during which only a handful of plague infections occurred in each month, before the onset of a second, more deadly phase from June 1666 until the last death in October 1666.

### (c) Informal description of transmission model

In order to investigate the routes of plague transmission in the Eyam outbreak, we created a purpose-built stochastic epidemiological model based on the results of the exploratory data analysis above. A closed population was assumed due to the effect of the quarantine and the exclusion of deaths from other causes and births during the outbreak. There is evidence that in some cases the quarantine was broken, notably by the Reverend Mompesson, whose children were sent away to safety in Yorkshire [28]. Additionally, it has been suggested that one of the reasons for the reduced death toll among wealthy families could be due to their having fled the area [33]. Only three cases of recovery from plague in Eyam are mentioned in the oral history, and none are recorded in the primary data sources [30]. As such, in accordance with previous studies, no recovery is allowed for in the model [34]. A separate analysis in which we considered that these three individuals had been infected and had recovered at the time suggested by oral tradition resulted in estimates for the transmission parameters that were not significantly different from the ones we inferred when no recovery was allowed.

**Figure 2.**
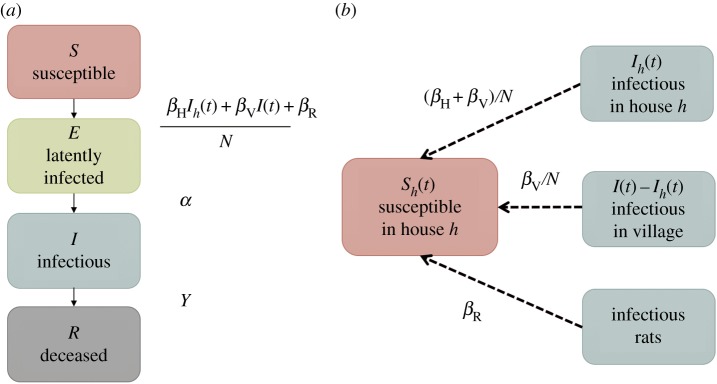
(*a*) Flow diagram of model compartments with rates of transition between infection states for an individual in house *h*. (*b*) Diagram showing routes of plague transmission to a susceptible individual in house *h*. (Online version in colour.)

Our model accounts for the possibility of both rodent-to-human and human-to-human transmission, as well as the known household structure [47]. Briefly, individuals are initially susceptible (S), become exposed (E), infectious (I) and finally removed through death (R) (SEIR model; figure 2*a*). Infection (transition from state S to E) happens through exposure from infected rodents, from other infected individuals in the household or elsewhere in the village (figure 2*b*). The five parameters of this model are thus the rate *β*_R_ of rodent-to-human transmission, the rate *β*_V_/*N* of transmission between humans who are not in the same household, the additional rate *β*_H_/*N* of transmission within households, the rate *α* at which infected individuals become infectious (transition from state E to I), and finally, the rate *γ* at which infectious individuals are removed (transition from state I to R). For a more detailed and formal description of the model, see the Material and methods section.

### (d) Analysis of transmission routes

Bayesian inference of the model parameters was performed using a Monte Carlo Markov chain (MCMC) algorithm. Data augmentation techniques [45] were used to account for the uncertainty in the time at which individuals became infected and infectious. Visual inspection of the trace plot and the prior and posterior densities of each parameter indicated good convergence and mixing (electronic supplementary material, figure S1), which was confirmed by the fact that when comparing independent runs the Gelman–Rubin statistic [48] was less than 1.1 for all parameters. For all parameters, informative posterior densities were obtained, despite the use of uninformative priors uniform from 0 to 100. Table 2 presents the posterior means, standard deviations and 95% credibility intervals for all model parameters. The latent phase of infection (state E in our model) was estimated to last on average 1/*α* = 5.6 days (95% credibility interval: [4.8, 6.3]) and the infectious phase (state I in our model) had a mean duration of 1/*γ* = 2.4 days (95% credibility interval: [2.1, 2.9]).

**Table 2.** Posterior mean, standard deviation (s.d.) and 95% credibility interval (CI) for model parameters under hypothesis *β*_H_ = *β*_V_ = 0, hypothesis *β*_H_ = 0 and under the full model.

	*β*_H_ = *β*_V_ = 0			*β*_H_ = 0			full model		
*Θ*	mean	95% CI	s.d.	mean	95% CI	s.d.	mean	95% CI	s.d.
*β*_H_	—	—	—	—	—	—	16.10	[10.53, 22.52]	3.06
*β*_V_	—	—	—	0.33	[0.27, 0.40]	0.03	0.29	[0.23, 0.35]	0.03
10^3^*β*_R_	0.96	[0.84, 1.07]	0.06	0.34	[0.25, 0.45]	0.05	0.31	[0.23, 0.41]	0.05
*α*	0.18	[0.16, 0.20]	0.01	0.18	[0.15, 0.20]	0.01	0.18	[0.16, 0.21]	0.01
*γ*	0.38	[0.33, 0.43]	0.03	0.41	[0.36, 0.47]	0.03	0.41	[0.35, 0.47]	0.03

A similar analysis was also performed assuming that transmission of plague did not occur from human to human (i.e. *β*_V_ = 0 and *β*_H_ = 0), but this hypothesis was decisively rejected by Bayesian model comparison using a reversible jump MCMC [49,50] (Bayes factor greater than 10^10^). The alternative hypothesis in which human-to-human transmission does happen but is not more frequent within households (i.e. *β*_V_ > 0 and *β*_H_ = 0) was also decisively rejected (Bayes factor greater than 10^10^). There is therefore conclusive evidence that human-to-human transmission played a role in the Eyam plague epidemic, and that the proximity of sharing a household increased transmission, which justifies the use of our model incorporating human-to-human transmission and household structure.

The expected proportion of total infections caused by rodent-to-human transmission versus human-to-human transmission was calculated, as well as the expected proportion of human-to-human transmission events that occurred inside the household as opposed to from the village at large (electronic supplementary material, figure S2). The model suggests that 73.0% of infections came from human-to-human transmission (95% credibility interval: [67.3%, 78.2%]), with the remaining 27.0% of infections caused by rodents (95% credibility interval: [21.8%, 32.7%]). Of the infections that came from human-to-human transmission the model predicts that 17.5% come from contact with infectious persons in the same household (95% credibility interval: [11.8%, 23.6%]), with the majority of 82.5% coming from contact with infectious persons in the rest of the village (95% credibility interval: [76.4%, 88.2%]). Transmission from an infectious to a susceptible individual happens at a rate (*β*_H_ + *β*_V_)/*β*_V_ = 56 times greater if the two individuals are in the same household compared with if they are not. This rate ratio was expected to be greater than one as a consequence of increased contact rate within households, and its high inferred value suggests that our model correctly captured interhuman transmission.

### (e) Seasonality effect

**Figure 3.**
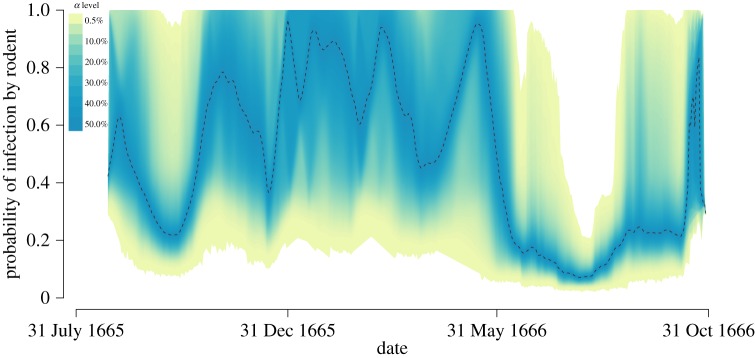
Probability that infection is caused by rodent-to-human transmission, with the shaded area representing the 99.5% credibility interval from 10 000 MCMC iterations. (Online version in colour.)

The probability that each observed infection was caused by rodents rather than interhuman transmission was plotted over the course of the epidemic (figure 3). During the colder months transmission from rodents played a relatively larger role, and there is a possibility that human-to-human transmission did not occur at all since the upper boundary of the 99.5% credibility interval reaches one. On the other hand, during the two peaks of the epidemic in October 1665 and June–August 1666 human-to-human transmission is the cause of most infections. However, because the data only span a year, it is not possible to conclude whether this pattern repeats itself with the alternation of cold and warm months.

To conclusively demonstrate a seasonality effect, it is therefore necessary to test whether such a phase of mostly rodent-driven transmission could happen in our model, which assumes that the transmission parameters are the same throughout the year and therefore does not account for seasonality. To this end, the real data were compared with simulated datasets using the same parameters as were inferred for the real data, also known as a posterior predictive distribution [51]. Although the simulated epidemics predict a similar number of deaths overall to the number actually observed, we find that the period during winter when very few infections were observed in Eyam is slightly outside of the simulated intervals (electronic supplementary material, figure S3). This suggests that there is a seasonality effect in the Eyam outbreak, consistent with general knowledge about the plague [2]. The seasonality of plague is usually explained by lower flea activity during colder months [19], but since little human-to-human transmission was observed in the winter (figure 3) an alternative or complementary explanation may be reduced human interactions during the cold season.

### (f) Infection risk factors

In order to test the effect of personal risk factors such as wealth, sex and age, posterior predictive distributions were constructed based on simulated epidemics using the same parameters as inferred for the Eyam dataset. This technique enables us to go beyond the properties of our model by capturing features of the data that are significantly different from the model expectation.

The question of whether household wealth affected the likelihood of contracting plague was investigated by comparing the observed proportion of plague victims that were from wealthy houses (those listed as charged on the hearth tax register) with the equivalent proportion from the simulated epidemics. There is significant evidence (*p*-value of less than 0.001) to suggest that people in wealthy houses were less likely to contract plague than those in poorer houses. In Eyam, only 20.2% of plague victims came from houses that appeared on the hearth tax register, whereas the simulated epidemics suggest with 99.9% probability that if the chances of contracting plague were independent of household wealth between 21.0 and 37.0% of the victims would be from wealthy houses. The differential in infection rates could perhaps be explained by better standards of cleanliness in wealthier households leading to fewer rodents and fewer human parasites, or, as has been suggested, by wealthier families fleeing the plague [33].

**Figure 4.**
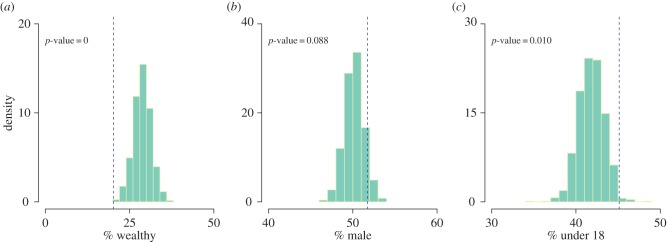
Histograms of proportion of plague victims that were (*a*) from wealthy households, (*b*) male and (*c*) under 18 years old male based on a thousand simulated epidemics using model parameters taken from the posterior distribution. The dotted lines show equivalent proportions observed in the Eyam data. (Online version in colour.)

There were slightly more men affected in the data relative to women, and even though this was found to be not statistically significant in the exploratory analysis, some previous studies have reported such an association between plague and men [52]. We therefore explored this hypothesis again by comparing the observed proportion of plague victims that were male with the equivalent proportion from the simulated epidemics. Figure 4 shows that there is not significant evidence in the Eyam epidemic to suggest that men were disproportionately more affected than women (*p* = 0.088). In total, 51.7% of plague victims were male, which is within the 99.9% posterior predictive interval [46.7%, 53.7%]. Previous analysis of the Eyam data has suggested that age could be a significant determining factor in the epidemic, with a higher death toll observed among younger adults compared with the very old or very young [32,37,39]. We therefore explored the effect of age by comparing the observed proportion of plague victims that were under 18 at the start of the epidemic with the equivalent proportion from the simulated epidemics. Figure 4 shows that there is significant evidence in the Eyam epidemic to suggest that children were disproportionately more affected than adults. In total, 45.1% of plague victims were under 18 (*p* = 0.010), which is significant; however, it is within the 99.9% posterior predictive interval [36.9%, 46.5%].

## 3. Discussion

Detailed information for both victims and survivors of the Eyam plague outbreak were combined with new epidemiological models and statistical methods of analysis to provide the most complete picture to date of the epidemiology of this famous tragedy. We found evidence for both rodent-to-human and human-to-human transmission routes, with these two routes accounting, respectively, for a quarter and three-quarters of all infection cases. It should be emphasized that under the formulation of the model, human-to-human transmission incorporates infection via vectors, such as the human flea *P. irritans* [21] and the human louse [20], and not only through aerosols caused by the relatively rare form of pulmonary plague [2]. The likely route of plague transmission varied over the course of the epidemic. Rodent-only transmission cannot be excluded during the colder months, as opposed to the two peaks of the epidemic in October 1665 and in June–October 1666 during which human-to-human transmission must have played a role in the spread of the disease (figure 3). This observation, combined with the fact that fewer cases occurred during the winter than would be expected without a seasonality effect (electronic supplementary material, figure S3), suggest a possible reduction in the rate of interhuman transmission during the cold season, possibly in conjunction with diminished rodent activity [19].

The role of the household structure was also found to be highly significant, to the extent that an infectious individual in the same household is almost 100 times more likely to transmit to a susceptible host compared with an infectious person living elsewhere in the village, which explains why so many members of the same families died in close succession [28,30,31] (figure 1). The presence of an infectious individual within the household is therefore a very important risk factor for contracting the disease. Gender was not an important factor in the epidemic, but on the other hand household wealth was confirmed to be an important determinant, with richer villagers that were liable for the hearth tax much less likely to die than poor villagers (figure 4). Adult age was also found to reduce the risk of catching the disease, and these two significantly protective factors could be interpreted in terms of a reduced rate of interactions with other humans, and therefore exposure to interhuman transmission. These risk factors were investigated using posterior predictive tests, and their effects were not included within the model.

The main limitation of the work presented here is that we have been focusing solely on a single, relatively small outbreak of the plague, and therefore that any conclusion drawn could be argued not to be necessarily representative of the plague in general. There are two main reasons for choosing the Eyam outbreak as a case study. First, exceptionally detailed information has been gathered from several historical documents by local historians [30,31,40] which together allow a full depiction of the inhabitants of Eyam at the time of the outbreak. Second, the conditions in which the outbreak unfolded with little evidence for entry or exit of individuals from the isolated village of Eyam, partly due to the famous quarantine, which greatly simplifies attempts to build an epidemiological model of the outbreak. The exact values of parameters such as the rate of rodent-to-human transmission or the rate of human-to-human transmission within and between households would probably be different if they could be estimated for other outbreaks in other settings, such as in the case of large urban epidemics such as the Great Plague of London in 1664–1666 [53] or the Marseilles Plague in 1720–1723 [54]. However, the mechanisms of spread are likely to have been the same, even if their role may have been different relative to one another. In particular, our results feed into the long ongoing debate about the role of interhuman transmission through human ectoparasites [21,24]. With the plague still being endemic in several countries of Africa and Latin America, this debate is not just of historical importance but also of contemporary relevance to help deal with this neglected tropical disease, which could someday become a worldwide public health priority again [11].

## 4. Material and methods

### (a) Model specification and notations

A stochastic SEIR model was adopted, taking into account the household structure of the data as well as the underlying epidemic process of plague. Disease status was described in four compartments: susceptible (S), latently infected (E), infectious (I) and removed (R) (figure 2*a*). All mathematical notations are summarized in the electronic supplementary material, table S2.

Villagers are initially susceptible to the disease and infected through contact with infected rodents or through contact with infectious people in their household or in the village at large. The model assumes that plague can be transmitted from rodents to humans at a constant contact rate of *β*_R_, and that human-to-human transmission can occur, most likely via ectoparasite vectors, or directly through aerosols in the case of pneumonic plague. The contact rate with infected villagers is assumed to be a constant *β*_V_/*N*, where *N* is the initial population size [55]. To account for the observed household structure of infection, an additional contact rate of *β*_H_/*N* is proposed to allow for the higher probability of transmission from infected members of the same household (figure 2*b*). Infected individuals are not immediately infectious and first pass through an exposed stage (E), at rate *α* per day before becoming infectious. The infectious stage is defined as the period of time during which infectious individuals can transmit the disease through contact with susceptibles. Finally, the infectious individuals are removed from the population, through death from plague, at rate *γ* per day.

It is assumed that death from plague occurred on the day prior to recorded burial, with exact times of death allocated to ensure a unique ordering of events. In household *h* of size *N_h_*, the date of death from plague for person 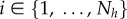 is denoted *ψ_h,i_*. The times of infection with plague, and the times when infected individuals become infectious themselves, are unknown, and are denoted *ϕ_h,i_* and *ν_h,i_*, respectively. For those villagers surviving the plague, we take 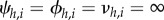. Data augmentation [45] is used to calculate the times of infection and becoming infectious, with the set of augmented data denoted 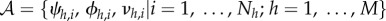.

Define 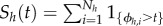, 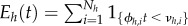, 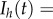 
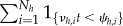
, 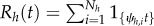 to be the number of susceptible, latently infected, infectious and removed (i.e. dead) persons in household *h* = 1, … , *M* at time *t*. Let 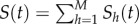, 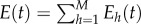, 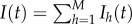, 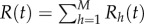 and 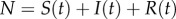.

### (b) Likelihood derivation

Denoting the set of model parameters as 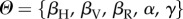, the joint probability of the observed data 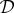, augmented data 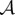 and parameters is4.1
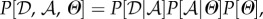
where 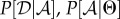 and 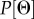 are referred to as the observation, transmission and prior levels, respectively [47].

The observation level of the model serves to ensure that the augmented data 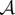 are consistent with the observed data 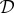. This is deemed to be the case when the period of infectiousness (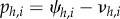) is shorter than the total period of infection (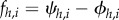); and the total period of infection is less than 30 days, where the maximum infection period before death has been chosen as a biologically realistic upper bound.4.2
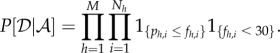


The transmission level describes plague transmission within each household, assuming the total infection and infectious periods 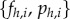 are known. For household *h*, the instantaneous rate of infection with plague at time *t* is4.3
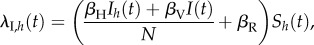


where *β*_V_ is the transmission rate of infection with plague from within the village; *β*_H_ is the additional rate of infection with plague from contact within the household and *β*_R_ is the transmission rate of infection due to contact with rodents.

The rate at which people in household *h* with latent infections become infectious is *λ*_E*,h*_(*t*), where *λ*_E*,h*_(*t*) = *αE_h_*(*t*) and *α* is the per-person rate of becoming infectious. Therefore, 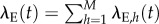 is the rate of latently infected people becoming infectious in the population as a whole.

The rate of death from plague in household *h* is denoted *λ*_D*,h*_(*t*), where *λ*_D*,h*_(*t*) = *γI_h_*(*t*) and *γ* is the rate of death from plague.

Let 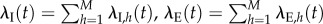 and 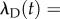 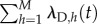 be the rates of infection, becoming infectious and death from plague in the population as a whole.

Let *τ* be the time to the next event of either type *I*, *E* or *D* in the population as a whole. Then *τ* ∼ Exp(*λ*(*t*)), where 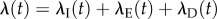.

If a total of *T* events happen over the course of the epidemic, then let the times at which those events occur be denoted *t*_1_, … , *t_T_*, where *t*_0_ = 0 is the time at which the process starts. Let *τ_i_* = *t_i_* − *t_i_*_−1_ be the inter-event times. Further, let 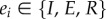 for *i* = 1, … , *T* be the observed events that occur, and let *h*_1_, … ,*h_T_* be the households in which those events occur.

The probability of the augmented data given the parameters is then4.4
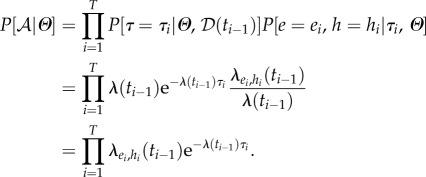


Uninformative prior distributions were assigned to the model parameters, and it was assumed that for 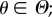 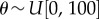, so *P*[*θ* = *x*] = 0.01.

### (c) Monte Carlo Markov chain methodology

MCMC methods were used to estimate the model parameters given epidemic data. A Markov chain was constructed such that its stationary distribution was 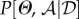, the posterior distribution of the model parameters and the augmented data given the observed data. The chain was started with augmented data that were consistent with the observed data. For each plague victim *i* in household *h* the initial length of the infection period *f_h,i_* was drawn from uniform distribution *U*[0, 30], and the length of the infectious period, *p_h,i_*, was drawn from uniform distribution *U*[0, *f_h,i_*].

The sampler performs single-component Metropolis–Hastings sampling. At each iteration, the algorithm proposes to update the model parameters in the sequential order *β*_H_, *β*_V_, *β*_R_, *α* and *γ*; then proposes to update each infection duration *f_h,i_* in turn, then finally proposes to update each duration of infectious period *p_h,i_* in turn. The parameters and augmented data are proposed from a normal distribution, with mean equal to the last accepted sample value, and standard deviation chosen to ensure efficient mixing of the Markov chain (electronic supplementary material, table S3). Reflecting boundaries are specified for each of the proposal distributions to ensure that the parameters and augmented data are biologically plausible and consistent with the observed data.

After a burn-in period of 5000 iterations, 20 000 iterations of each model were performed and thinned by a factor of two to obtain a sample of 10 000 values from the posterior distribution. The convergence of the MCMC was assessed by examining trace plots of the sampled parameters, and then confirmed using the Gelman–Rubin criterion (GRC) [48]. Five chains with over-dispersed starting parameters were run for each model hypothesis. The GRC was estimated for each parameter and for the log-likelihood, with GRC < 1.1 being taken as confirmation of convergence.

### (d) Model comparison, simulation and assessment

In order to determine whether human-to-human transmission played a role in the Eyam epidemic—and, if so, to what extent was household structure a determinant—we used Bayesian model comparison [56]. First, we compared a model with no human-to-human transmission (i.e. *β*_H_ = *β*_V_ = 0) versus a model with no additional risk for transmission within the household (i.e. *β*_H_ = 0). Second, we compared a model with no additional risk for transmission within the household (i.e. *β*_H_ *=* 0) versus the full model described above. Each of these two comparisons was performed using a reversible jump MCMC [49,50], which was similar to the MCMC algorithm described above except for the addition of reversible jumps proposing to set the relevant parameter to zero and back. The validity of the reversible jump algorithms was tested using simulated data, and in particular when the smaller models were used for simulation, the smaller models were correctly selected. However, application to the real dataset always resulted in the larger of the two models being used at every MCMC iteration. Since the proportion of sampling from the compared models reflects the posterior odds ratio, which is equal to the odds ratio times the Bayes factor, and that the smaller models were not sampled even when the prior odds ratio was increased up to 10^10^ in favour of the smaller models [57,58], we conclude that the Bayes factor is greater than 10^10^ in favour of the larger models for both comparisons.

In order to simulate data under our model, an epidemic simulator was built as follows. Given the Eyam household structure and input parameters *Θ*, the time until the first event was drawn from an exponential distribution with parameter *λ*(*t*). The type of event to occur (i.e. an infection, becoming infectious or a death) was determined by sampling 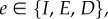 where 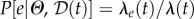. Finally, the household in which the event occurred was determined by sampling 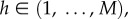 where 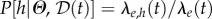. The state of the epidemic was updated and the process repeated until no infected individuals remained in the population and more than 350 days had elapsed.

Ten thousand epidemics were simulated using the same household structure as Eyam and known parameters *Θ*, drawn from the posterior distribution derived from the Eyam epidemic, in order to build the posterior predictive distributions [51] required to test the effect of seasonality and personal risk factors.

## Supplementary Material

Supplementary Table 1

## Supplementary Material

Supplementary Material

## Data accessibility

All data used in this study are available in the electronic supplementary material, table S1.
