# Supplementary material for: Epidemiological analysis of the Eyam plague outbreak of 1665–1666
Source: Proc Biol Sci. 2016 May 11;283(1830):20160618. doi: 10.1098/rspb.2016.0618 (PMC4874723; doi:10.1098/rspb.2016.0618)
Supplement: Supplementary Material [file rspb20160618supp2.pdf]

SEE SEPARATE EXCEL FILE

Table S1. Data used in this study

Augmented data		
$\mathcal{A}$	Description	
$\phi_{h,i}$	time of infection for i in h	
$\nu_{h,i}$	time of becoming infectious for i in h	
$\psi_{h,i}$	time of death (observed) for i in h	
Model parameters		
Θ	Description	
$\frac{\beta_H}{N}$	additional human-to-human transmission rate within household (per person, per day)	
$\frac{\beta_V}{N}$	human-to-human transmission rate within village (per person, per day)	
β_R	rodent-to-human transmission rate	
α	rate of becoming infectious (per day)	
γ	rate of death from plague (per day)	
Calculated items		
	Description	Formula
$f_{h,i}$	length of infection period for i in h	$\psi_{h,i} - \phi_{h,i}$
$p_{h,i}$	length of infectious period for i in h	$\psi_{h,i} - \nu_{h,i}$
$S_h(t)$	number susceptible at t in h	$\sum_{i=1}^{N_h} 1_{\{\phi_{h,i} > t\}}$
$E_h(t)$	number latently infected at t in h	$\sum_{i=1}^{N_h} 1_{\{\phi_{h,i} \leqslant t < \nu_{h,i}\}}$
$I_h(t)$	number infectious at t in h	$\sum_{i=1}^{N_h} 1_{\{\nu_{h,i} \leqslant t < \psi_{h,i}\}}$
$R_h(t)$	number deceased at t in h	$\sum_{i=1}^{N_h} 1_{\{\psi_{h,i} \leqslant t\}}$
$\lambda_{I,h}(t)$	instantaneous rate of infection at t in h	$\frac{1}{N}[(\beta_H I_h(t) + \beta_V I(t)) + \beta_R]S_h(t)$
$\lambda_{E,h}(t)$	rate of becoming infectious at t in h	$\alpha E_h(t)$
$\lambda_{D,h}(t)$	rate of death from plague at t in h	$\gamma I_h(t)$

Table S2. Notation used in the model description for individuals $i = 1, ..., N_h$ in households $h = 1, ..., M$ at time t

	Parameter	Prior distribution	Proposal distribution	σ	Min	Max
Θ	$\beta_H^{(k)}$	$U[0,100)$	$N(\beta_H^{(k-1)},\sigma^2)$	5	0	100
	$\beta_V^{(k)}$	$U[0,100)$	$N(\beta_V^{(k-1)},\sigma^2)$	0.5	0	100
	$\beta_R^{(k)}$	$U[0,100)$	$N(\beta_R^{(k-1)},\sigma^2)$	10^{-4}	0	100
	$\alpha^{(k)}$	$U[0,100)$	$N(\alpha^{(k-1)},\sigma^2)$	0.3	0	100
	$\gamma^{(k)}$	$U[0,100)$	$N(\gamma^{(k-1)},\sigma^2)$	0.5	0	100
$\mathcal{A}$	$f_{h,i}^{(k)}$	n/a	$N(f_{h,i}^{(k-1)},\sigma^2)$	5	$p_{h,i}^{(k-1)}$	30
	$p_{h,i}^{(k)}$	n/a	$N(p_{h,i}^{(k-1)},\sigma^2)$	5	0	$f_{h,i}^{(k)}$

Table S3. Prior and proposal distributions for parameters and augmented data at iteration k for persons $i = 1, ..., N_h$ in households $h = 1, ..., M$ at time t

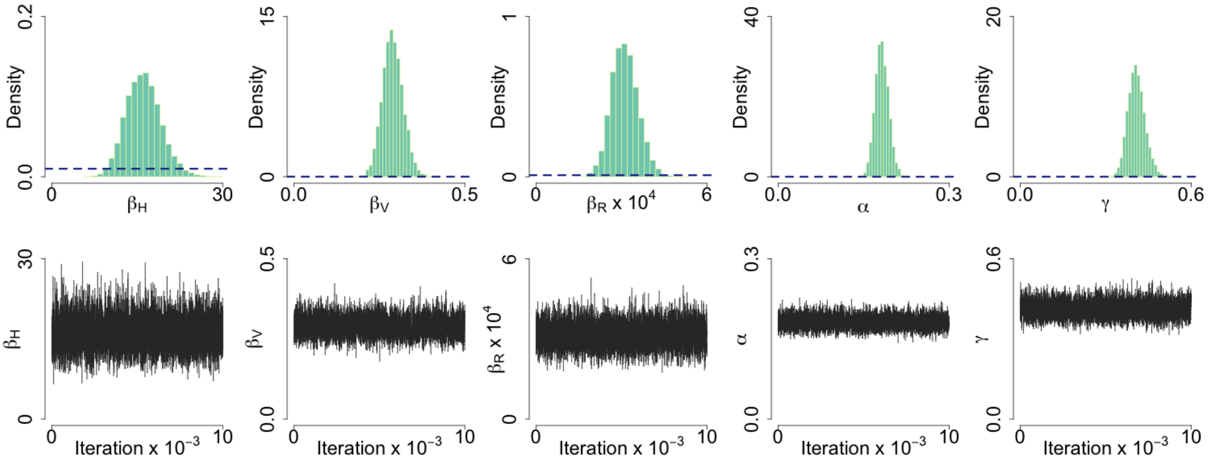

Figure S1. The top row shows histograms of posterior parameter values under our model. Blue dashed lines show the prior density. The bottom row shows MCMC trace plots of the parameter values.

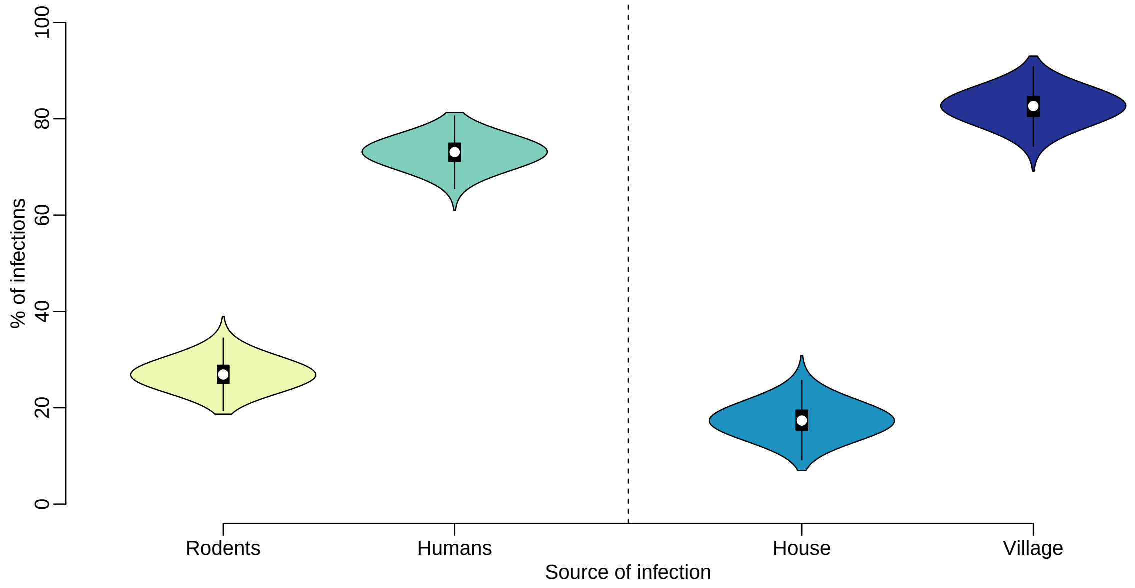

Figure S2. Left: expected proportion of total infections caused by contact with rodents vs. humans. Right: Expected proportion of human-to-human infections caught within household vs. in village.

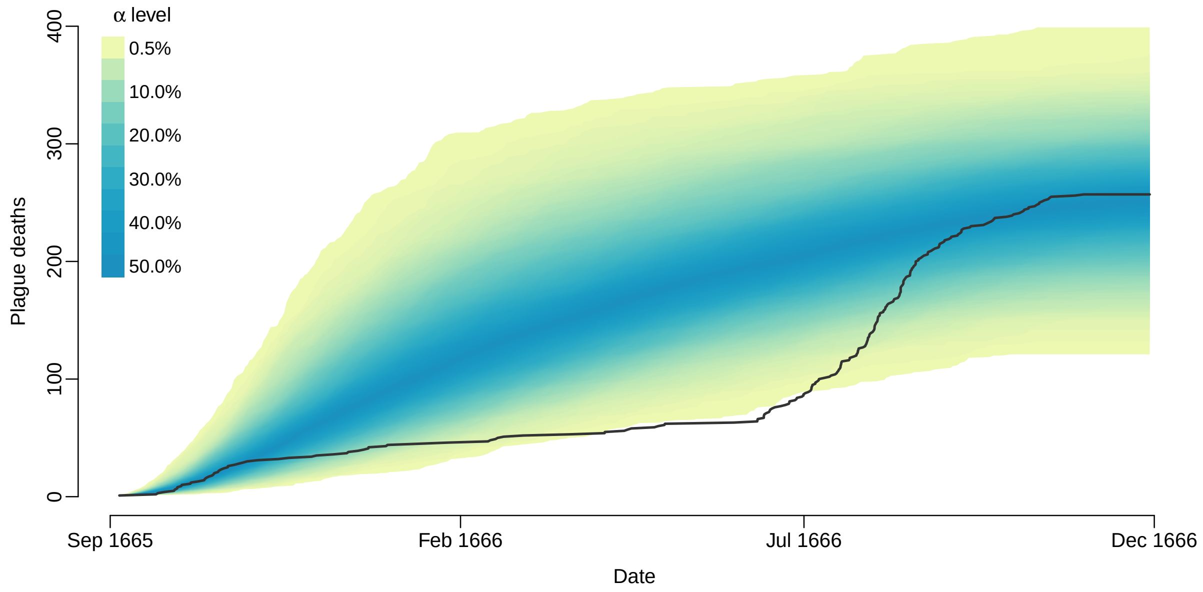

Figure S3. Comparison of simulated and observed deaths in Eyam due to plague. The dotted line represents observed deaths, and the shaded area the 99.5% posterior predictive interval from a thousand simulated epidemics using parameters drawn from their posterior distributions.
